# Mucinous components assessed by magnetic resonance imaging in primary rectal cancer tissue before and after chemoradiotherapy and tumor response

**DOI:** 10.1007/s00384-018-3047-1

**Published:** 2018-04-26

**Authors:** Hiroshi Miyakita, Sotaro Sadahiro, Takashi Ogimi, Gota Saito, Kazutake Okada, Akira Tanaka, Toshiyuki Suzuki, Hiroshi Kajiwara, Hiroshi Yamamuro, Takeshi Akiba

**Affiliations:** 1 Department of Surgery, Tokai University School of Medicine, 143 Shimokasuya, Isehara, Kanagawa 259-1193 Japan; 2 Department of Pathology, Tokai University School of Medicine, Tokyo, Japan; 3 Department of Radiology, Tokai University School of Medicine, Tokyo, Japan

**Keywords:** Rectal cancer, Chemoradiotherapy, Mucin pool, Mucinous carcinoma, Pathological response, Tumor shrinkage

## Abstract

**Background:**

Mucinous rectal carcinoma has been reported to have a lower survival rate and a poorer histologic response to chemoradiotherapy(CRT). Magnetic resonance imaging (MRI) can accurately evaluate the amount of mucin pools (MP) in primary cancer tissue. We compared the degree of MP on MRI before and after CRT with the histologic findings of resected specimens to investigate the predictors of response to CRT.

**Methods:**

The study group comprised 205 patients with rectal adenocarcinoma who received preoperative CRT. MPs were measured on MRI before and after CRT and in resected specimens. The degree of MP was classified into five classes according to the MP area ratio: 0%, class I; 1 to 19%, class II; 20 to 49%, class III; and 50% or higher, class IV.

**Results:**

The degree of MP on MRI was largely unchanged after CRT; however, the MP on MRI after CRT was underestimated in 26.3% of patients as compared with that in resected specimens. A pathological complete response was obtained in patients who initially had no MP or had an MP ratio of less than 20%. The tumor volume was significantly greater, and the rates of tumor shrinkage and T downstaging were significantly lower in patients who had an MP area ratio of 20% or higher before CRT than in those who had an MP area ratio of less than 20%.

**Conclusions:**

The MP area ratio measured on MRI before treatment was closely associated with the response to CRT and is a potentially useful predictor of treatment response.

## Introduction

Mucinous carcinoma is a characteristic histologic type of colorectal cancer that has been reported to account for 8 to 10%, and 17% [1] and 7.1% [2] of rectal cancers. In patients with locally advanced rectal cancer, the standard treatment is CRT followed by surgery. Mucinous carcinoma has been reported to be associated with a lower survival rate [1, 2], a poorer histologic response to preoperative CRT, and a lower pathological complete response (pCR) rate than non-mucinous carcinoma (differentiated adenocarcinoma) [3, 4].

Mucinous carcinoma is difficult to diagnose on biopsy, and the rate of accurate diagnosis has been reported to be 5 to 8% [3, 4]. Magnetic resonance imaging (MRI) can be used to accurately evaluate the amount of MP in primary cancer tissue [5, 6].

CRT has been reported to induce the formation of mucin pools (MP) in cancer tissue [3, 4]. We therefore hypothesized that the amount of MP in primary cancer tissue evaluated on MRI before CRT is closely related to the response to CRT. We compared the degree of MP on MRI findings before and after CRT with the histologic findings of the resected specimens to investigate predictors of the response to CRT.

## Subjects and methods

### Subjects

The study group comprised 205 patients with clinical T3-T4, any-N adenocarcinoma of the middle and lower rectum who received radiotherapy (40 to 45 Gy) and concurrent chemotherapy with oral uracil/tegafur plus leucovorin or with S-1 from January 2006 through December 2014 [7, 8]. Surgical resection was performed within 6 to 8 weeks after the completion of CRT.

### Evaluation of MP

The degree of MP was evaluated on MRI in accordance with the reports of Hussain [5] and Kim [6]. The area ratio of MP on the maximum cut surface of the primary tissue was calculated. A 1.5-T MRI system with a surface coil was used. Imaging analysis was performed using a Digital Imaging and Communications in Medicine (DICOM) viewer (SDS viewer, Ver. 8.0.4.4, TechMatrix Corporation, Tokyo, Japan).

We histologically evaluated the most predominant histologic type on specimens stained with hematoxylin and eosin and assessed the presence or absence of MP and the MP area ratio on the maximum cut surface of the tumors. The MP area ratio was separately evaluated by two physicians (HM and SS), and the mean value was adopted. The degree of MP was classified into the following four levels according to the MP area ratio: MP 0%, class I; MP 1 to 19%, class II; MP 20 to 49%, class III; and MP 50% or higher, class IV.

### Evaluation of tumor shrinkage rate and histologic regression

The tumor shrinkage rate was calculated on the basis of the tumor size evaluated on MRI before and after CRT [9]. Histologic regression was classified according to the tumor regression grade (TRG) [10]. TRG was classified as Grade 1 (complete regression), Grade 2 (presence of rare residual cancer cells), Grade 3 (increased number of residual cancer cells), Grade 4 (residual cancer outgrowing fibrosis), or Grade 5 (absence of regression change). A TRG of 1 and 2 was defined as marked regression.

### Statistical analyses

Groups were compared with the use of Fisher’s exact test or the chi-square test for categorical variables and the Mann-Whitney *U* test or the Kruskal-Wallis test for continuous variables. The correspondence rates between the classes of MP evaluated on MRI before CRT, MRI after CRT, and in resected specimens were analyzed with the kappa coefficient (k). In all statistical analyses, a two-sided value of *p* < 0.05 was considered to indicate statistical significance. Statistical calculations were performed using JMP version 11 software (SAS Institute Inc., Cary, NC, USA).

This study was approved by the institutional review board of our University (08R-032), and all patients provided their written informed consent.

## Results

### Patient characteristics

The tumor was located in the middle rectum in 82 patients and the lower rectum in 123 patients. The pathologic stage after CRT (ypStage) was ypStage 0 (including pCR) in 31 patients (15.1%), ypStage I in 66 patients (32.2%), ypStage II in 62 patients (30.2%), ypStage III in 37 patients (18.5%), and ypStage IV in nine patients (4.4%).

### Evaluation of MP on MRI after CRT and evaluation of MP in resected specimens

MPs were histologically found in 63 (30.7%) of the 205 patients. Class III and IV MPs, in which MPs accounted for at least 20% of the tumor volume, were found in 20 patients (9.7%).

The class of MP was the same on MRI and resected specimens in 151 patients (73.7%). In the other 54 patients (26.3%), the class of MP was consistently higher in resected specimens and was not lower in resected specimens in any patient. MP was confirmed histologically in 42 (20.5%) of the 184 patients with no MP on MRI. The correspondence rate of the MP between the MRI findings and histology was poor (*k* = 0.023).

### Comparison of MP evaluated on MRI before and after CRT

The classes of MP before and after CRT were the same in 193 patients (94.1%). The class of MP increased after CRT in four patients (1.9%) and decreased after CRT in eight patients (3.9%). The degree of MP evaluated on MRI was largely unchanged between before and after CRT. The correspondence rate of the MRI findings was moderate (*k* = 0.50).

### Relations of the class of MP before CRT to tumor volume, downstaging, tumor shrinkage rate, and histologic regression

The MP area ratio was measured on MRI before treatment, and 20% was regarded as the cutoff value. The tumor volumes, shrinkage rates, T and N downstaging, and pCR rates are shown in Table 1. Patients with an MP of < 20% had significantly smaller tumors (*p* = 0.001), a significantly higher shrinkage rate (*p* = 0.002), and a significantly higher T downstaging rate (*p* = 0.047) than did patients with an MP of ≥ 20%. All of 25 patients with pCR had an MP of < 20%, with no significant difference in the rates of pCR or marked regression.

**Table 1:** Tumor volumes, tumor shrinkage rates, and histologic response according to the Class of MP measured on MRI before starting CRT

	MP < 20%	MP ≧ 20%	*p* value
	*n* = 194 (%)	*n* = 11 (%)	
Tumor mean volume (cm^3^)			
Mean ± SD	11.8 ± 13.1	34.6 ± 26.5	0.001*
Range	0.48–73.5	0.69–81.2	
Shrinkage rate			
Mean ± SD	0.74 ± 0.17	0.56 ± 0.27	0.002*
Range	0.17–1.00	0–0.91	
T factor			
Down stage	112 (58)	3 (27)	0.047†
N factor			
Down stage	82 (49)	4 (44)	0.699†
TRG‡			
1, 2 (marked regression)	120 (62)	7 (64)	0.906†
Pathological evaluation			
pCR§	25 (13)	0	0.204†

*Mann–Whitney *U* test

† Fisher’s exact test

‡Tumor regression grade

§Pathological complete response

## Discussion

As for the diagnosis of MP on MRI, T2-weighted fast spin-echo imaging has been reported to be useful for the diagnosis of mucinous carcinoma in the primary tissue [5]. Kim et al. reported that mucinous carcinoma was correctly diagnosed in 97% of patients [6]. In our study, however, the evaluation of MP on MRI after CRT was consistent with the histological evaluation of resected specimens in 73.7% of the patients, and MP area ratio was underestimated in 26.3% of the patients. These findings are most likely attributed to the fact that all patients in our study received CRT, whereas the patients in the study of Kim et al. did not receive CRT.

Sclafani F et al. reported that the rate of concordance between the TRG predicted on MRI and the TRG evaluated on resected specimens was only 71% in patients who received CRT. A pCR was correctly diagnosed on MRI in only 74.4% of the patients, indicating that the rate of correctly diagnosis after CRT is not high [11]. In our study, two of 11 patients with an MP area ratio of ≥ 50% (class IV) on resected specimens were evaluated to have class II MP (MP, 1 to 19%) on MRI, suggesting that the diagnostic accuracy of MP on MRI decreases after CRT.

In a recent study, patients were randomly divided into two groups according to the interval between the completion of radiation and surgery: 7 vs. 11 weeks. The colloid response rate was significantly higher in the 11-week group (31.4%) than in the 7-week group (19.4%, *p* = 0.044) [12]. These findings suggest that CRT induces the formation of MP in some patients. However, when MRI findings before CRT were compared with MRI findings after CRT, the MP area ratio on MRI was evaluated to have increased in only 2% of the patients. We therefore considered that evaluation of the degree of MP before CRT might be most strongly related to the response to CRT.

Patients with an MP of ≥ 20% before CRT had a significantly greater tumor volume, a significantly lower shrinkage rate, and a significantly lower T downstaging rate. None of the patients with an MP of ≥ 20% before CRT had a pCR. These findings are consistent with the results of previous studies showing that mucinous carcinomas are associated with a larger tumor volume and a poorer response to CRT [3, 4].

Mucinous carcinoma is difficult to diagnose on biopsy [3, 4]. In our series, mucinous carcinoma was diagnosed in only one (9%) of 11 patients with an MP of ≥ 20% on MRI before CRT. Therefore, the diagnosis of MP in cancer tissue by MRI before treatment is considered useful for the prediction of treatment response and determination of the treatment strategy.

## Conclusions

Patients who had an MP area ratio of ≥ 20% on MRI before treatment had large tumors and a poor response to CRT. The MP area ratio measured on MRI before treatment is useful for the prediction of treatment response and determination of the treatment strategy.
